# Next-generation sequencing to monitor the spread of antimicrobial resistance

**DOI:** 10.1186/s13073-017-0461-x

**Published:** 2017-07-25

**Authors:** Michael Otto

**Affiliations:** Pathogen Molecular Genetics Section, Laboratory of Bacteriology, National Institute of Allergy and Infectious Diseases, U.S. National Institutes of Health, Bethesda, MD 20814 USA

**Keywords:** Antibiotic resistance, ESBL, *Escherichia coli*, MRSA, Next-generation sequencing, *Staphylococcus aureus*

## Abstract

Next-generation sequencing is increasingly being used to monitor current and historic events related to the emergence and spread of antimicrobial resistance. In a recent publication, researchers analyzed the rise of methicillin-resistant *Staphylococcus aureus* in the 1960s, emphasizing that adaptations conferring antibiotic resistance might pre-date the introduction of novel antibiotic derivatives. Other researchers have evaluated the role of transmission within a healthcare network, using the example of extended-spectrum beta-lactamase-resistant *Escherichia coli*.

Please see related Genome Biology Research article: www.dx.doi.org/10.1186/s13059-017-1252-9 and Genome Medicine Research article: www.dx.doi.org/10.1186/s13073-017-0457-6

## Antimicrobial resistance as an enduring public health threat

Antimicrobial resistance (AMR) is a major cause of morbidity and mortality on a global scale. In the US alone, the Center for Disease Control estimated the yearly number of deaths due to AMR to be at least 23,000 [1]. Exact global data are not available, but estimates put the current death toll at approximately 700,000 and the estimated annual number of deaths in 2050, if the case policies and strategies to combat AMR are not changed, at a staggering 10 million [2]. That death toll increases even further if one considers AMR-related conditions such as *Clostridium difficile* infections that can arise after the destruction of the intestinal microbiota by prolonged antibiotic therapy. Globally, most AMR-related deaths are caused by AMR in malaria, tuberculosis, and HIV, whereas, in highly developed countries, hospital-associated infections with methicillin-resistant *Staphylococcus aureus* (MRSA) and emerging pan-resistance in a series of Gram-negative bacteria, including Enterobacteriaceae with extended-spectrum beta-lactamase (ESBL) resistance, are the predominant problems. Beta-lactams, which include penicillin, all target bacterial cell wall synthesis. They are among the most important and most frequently prescribed antibiotics. After the introduction of penicillin into clinical use in the 1940s, strains that contained the genetic information to produce the enzyme beta-lactamase, which destroys the beta-lactam ring that is characteristic for that antibiotic class, spread globally at an immense pace [3].

In addition to the search for novel antibiotics, vaccines, and alternative drugs, infection control and surveillance measures represent an important part of the multi-pronged approach of public health systems, which researchers and the pharmaceutical industry need to follow to overcome the threat of AMR. For example, improved infection control and surveillance are believed to have caused the decline of MRSA infections observed in US hospitals over the past decade [4]. To understand and be prepared for emerging AMR threats, it is imperative to monitor the rise and spread of resistant pathogenic strains and lineages. Previously, typing schemes such as multi-locus sequence typing were used for that purpose, but the more-recent advances in DNA sequencing technology, offering broadly available and inexpensive next-generation sequencing, have opened the possibility to monitor AMR epidemiology in unprecedented detail. Using next-generation sequencing, two UK-based research groups recently addressed the epidemiology of beta-lactam resistance in ESBL *Escherichia coli* and MRSA.

## Monitoring transmission and reservoirs of AMR strains

In a study by Brodrick and colleagues, published recently in *Genome Medicine*, researchers analyzed ESBL *E. coli* in patients in long-term care facilities (LTCFs) in the UK [5]. *E. coli* is a major cause of urinary tract infections (UTIs), and infection with ESBL *E. coli* is associated with increased hospital stay, costs, and mortality. The uropathogenic *E. coli* lineage of sequence type (ST) 131 in particular is a widely disseminated cause of UTIs. Patients in LTCFs are known to carry ESBL-producing bacteria at a strongly increased frequency in comparison with the general population, owing to risk factors such as old age, urinary catheterization, and frequent use of antibiotics [6], but studies addressing AMR in LTCFs, especially using highly discriminative whole-genome sequencing (WGS), are still rare.

Using WGS of ESBL isolates from LTCF patients, but also from nearby and more distant UK hospitals, Brodrick and colleagues found that most of the LTCF isolates clustered together in a phylogenetic analysis, indicating a local lineage pre-dating the study. Furthermore, they found similarity to the isolates obtained from a nearby hospital, highlighting the role of regional transmission in the spread of resistant strains. Moreover, they identified distinct lineages within the LTCF samples, suggesting independent transmission into the LTCF, and detected within-host evolution. Finally, patients who had ESBL *E. coli* had received significantly more antibiotics in the year before the study. These outcomes of the WGS approach of the study fit well with what we would expect, inasmuch as they verify our notions about the role of transmission of resistant isolates in the healthcare network and the role of antibiotic use in selecting for antibiotic-resistant strains. Notably, they emphasize the power of next-generation sequencing for AMR surveillance and suggest that also other antibiotic-resistant pathogens and healthcare settings could benefit from such approaches.

## A new outlook on the evolutionary origins of MRSA

In a new study in *Genome Biology*, Harkins and colleagues analyzed the origins of MRSA, which emerged in the UK in the early 1960s [7]. In the late 1950s, beta-lactamase-mediated resistance to penicillin, once claimed to be a ‘miracle drug’, prompted the development of methicillin as a penicillin derivative to overcome this beta-lactamase sensitivity. Methicillin was introduced into clinical use in 1959, but, one year later, three methicillin-resistant *S. aureus* isolates of ST250 from a London hospital could already be detected during a nationwide screen for MRSA in the UK. Methicillin resistance in *S. aureus* is commonly assumed to have originated from horizontal transfer of the methicillin-resistance-encoding staphylococcal cassette chromosome (SCC) *mec* mobile genetic element from coagulase-negative staphylococci [8]. It is also commonly believed that it was the selective pressure due to the use of methicillin that caused the spread of this MRSA clone, after it had acquired the SCC*mec* element.

By contrast, Harkins and colleagues present evidence indicating that MRSA pre-dated the introduction of methicillin into clinical use, and the assumed selective pressure due to methicillin prescription, by more than a decade. Their WGS data of historical MRSA isolates also suggest that ST250 MRSA arose in one event of horizontal SCC*mec* transfer, which was then fixed in the population owing to a mutation in the recombinase gene, rendering the recombinase dysfunctional and precluding excision.

However, these data do not mean that the basic notion that antibiotic use drives the emergence of resistant clones is incorrect. Rather, one should take into consideration that the penicillin-binding protein 2a (PBP2a), which is the protein product of the resistance gene *mecA* within SCC*mec*, confers resistance not only to methicillin but to beta-lactam antibiotics, including penicillin, in general. Thus, the main message of the Harkins et al. study is that resistance to novel antibiotics that are merely derivatives of other antibiotics already in use might be preexisting owing to overlapping substrate specificity of the respective resistance mechanisms. In other words, resistance to penicillin, not methicillin, drove the emergence of MRSA.

These novel insights fit nicely with known epidemiological facts surrounding the emergence of MRSA, namely the lack of selective pressure at the beginning of methicillin use, and the structural relatedness of penicillin and methicillin, making it likely that the resistance mechanisms are also similar. However, before the use of WGS, the findings of Harkins and colleagues could not have been achieved despite the substantial research efforts that went into deciphering the events associated with the origin of MRSA. It is also important to note that collections of MRSA strains in the UK were kept in virtually untouched forms, without which this retrospective study would not have been possible.

An important lesson to be learned from the Harkins and colleagues’ study is that derivatization of existing antibiotics might not represent the best way to counter antimicrobial resistance—this emphasizes the need for truly novel antibiotics, which are, however, hard to find, and alternative strategies such as antivirulence drugs [9] and vaccines [10]. Furthermore, although the WGS data pinpointing the origin of SCC*mec* in the Harkin et al. study support their main mechanistic hypothesis, it will be interesting to verify in the future by experimental analysis the relative contribution of *mecA* to penicillin resistance in the historical strains.

## Concluding remarks

Next-generation sequencing approaches, as taken by Brodrick et al. and Harkins et al., represent a key advance in our efforts to track and understand the spread of AMR, inasmuch as they are able to produce previously unavailable detailed genetic evidence for hitherto mostly hypothetical epidemiological scenarios. However, to fully comprehend the rise of antibiotic resistance, it will be necessary to use a multi-pronged approach combining epidemiological, in silico, and experimental research.

## Abbreviations

AMR: Antimicrobial resistance
ESBL: Extended-spectrum beta-lactamase
LTCF: Long-term care facility
MRSA: Methicillin-resistant *Staphylococcus aureus*
SCC: Staphylococcal cassette chromosome
ST: Sequence type
UTI: Urinary tract infection
WGS: Whole-genome sequencing

## References

[1] Centers for Disease Control and Prevention. Antibiotic Resistance Threats in the United States, 2013. https://www.cdc.gov/drugresistance/threat-report-2013/index.html. Accessed 7/2/2017.

[2] Review on antimicrobial resistance. Antimicrobial resistance: tackling a crisis for the health and wealth of nations. O’Neill J editor. UK Government; 2014. https://amr-review.org/sites/default/files/AMR%20Review%20Paper%20-%20Tackling%20a%20crisis%20for%20the%20health%20and%20wealth%20of%20nations_1.pdf. Accessed 7/2/2017.

[3] Barber M, Rozwadowska-Dowzenko M (1948). Infection by penicillin-resistant staphylococci. Lancet.

[4] Dantes R, Mu Y, Belflower R, Aragon D, Dumyati G, Harrison LH (2013). National burden of invasive methicillin-resistant *Staphylococcus aureus* infections, United States, 2011. JAMA Intern Med.

[5] Brodrick HJ, Raven KE, Kallonen T, Jamrozy D, Blane B, Brown NM, et al. Longitudinal genomic surveillance of multidrug-resistant *Escherichia coli* carriage in a long-term care facility in the United Kingdom. Genome Med. 2017; doi:10.1186/s13073-017-0457-6. 10.1186/s13073-017-0457-6 PMC5525225 28738847

[6] Ludden C, Cormican M, Vellinga A, Johnson JR, Austin B, Morris D (2015). Colonisation with ESBL-producing and carbapenemase-producing Enterobacteriaceae, vancomycin-resistant enterococci, and meticillin-resistant *Staphylococcus aureus* in a long-term care facility over one year. BMC Infect Dis.

[7] Harkins CP, Pichon B, Doumith M, Parkhill J, Westh H, Tomasz A, et al. Methicillin resistant *Staphylococcus aureus* emerged long before the introduction of methicillin into clinical practice. Genome Biol. 2017; doi:10.1186/s13059-017-1252-9. 10.1186/s13059-017-1252-9 PMC5517843 28724393

[8] Otto M (2013). Coagulase-negative staphylococci as reservoirs of genes facilitating MRSA infection: Staphylococcal commensal species such as *Staphylococcus epidermidis* are being recognized as important sources of genes promoting MRSA colonization and virulence. Bioessays.

[9] Dickey SW, Cheung GYC, Otto M (2017). Different drugs for bad bugs: antivirulence strategies in the age of antibiotic resistance. Nat Rev Drug Discov.

[10] Schaffer AC, Lee JC (2009). Staphylococcal vaccines and immunotherapies. Infect Dis Clin North Am.

